# Sexual Dysfunction in Patients With Urinary Bladder Stones but no Bladder Outlet Obstruction

**DOI:** 10.3389/fmed.2021.704360

**Published:** 2021-09-07

**Authors:** Zhi-Cheng Gong, Zhi-Liang Wu, Yao-An Wen, Jie-Peng Zou, Xisheng Wang, Xiaoyan Leng, Anthony J. Bleyer, Chunhua Deng, Michael P. Feloney, Yuanyuan Zhang, Shan-Chao Zhao

**Affiliations:** ^1^Department of Urology, Nanfang Hospital, The First School of Clinical Medicine, Southern Medical University, Guangzhou, China; ^2^Department of Urology, Xiang'an Hospital of Xiamen University, Xiamen, China; ^3^Department of Urology, Dongfeng Zhongshan People's Hospital, Zhongshan, China; ^4^Department of Urology, Fujian Medical University Union Hospital, Fuzhou, China; ^5^Department of Urology, Shenzhen Longhua New District Central Hospital, Shenzhen, China; ^6^Public Health Sciences, Department of Biostatistics, Wake Forest University School of Medicine, Winston-Salem, NC, United States; ^7^Section on Nephrology, Wake Forest University School of Medicine, Winston-Salem, NC, United States; ^8^Department of Urology, The First Affiliated Hospital, Sun Yat-sen University, Guangzhou, China; ^9^Department of Urology, School of Medicine, Creighton University, Omaha, NE, United States; ^10^Wake Forest Institute for Regenerative Medicine, Wake Forest University School of Medicine, Winston-Salem, NC, United States; ^11^Department of Urology, The Third Affiliated Hospital, Southern Medical University, Guangzhou, China

**Keywords:** sexual dysfunction, bladder stones, lower urinary tract symptoms, surgical treatment, benign prostatic hyperplasia

## Abstract

**Objective:** To explore the correlates of sexual dysfunction and lower urinary tract symptoms (LUTS) in male patients with urinary bladder stones and to determine the effect of stone extraction on recovery of sexual function.

**Materials and Methods:** A total of 87 male patients with primary bladder stones were studied from January 2015 to May 2016. All patients underwent pneumatic lithotripsy for bladder stones. Sexual dysfunction was assessed based on sexual function assessment scales. The relationship of bladder stones with sexual dysfunction or LUTS was assessed using a two-sample *t*-test. Postoperative improvement of sexual function was assessed by repeated measures Analysis of Variance (ANOVA).

**Results:** Forty-one patients had primary bladder stones and 46 had secondary stones from the kidneys. Eighty-three of 87 patients (95%) had sexual dysfunction; 79 patients (91%) had both sexual dysfunction and LUTS. There was a significant association between bladder stones and sexual dysfunction, between sexual dysfunction and LUTS, and between bladder stone and LUTS (*p* < 0.05). There was no significant association between the course of illness, size and number of bladder stones, or urinary tract infection with sexual function (*p* > 0.05). In addition, among 83 patients with both bladder stone and sexual dysfunction, 61 patients (73%) had benign prostatic hyperplasia (BPH) and 22 patients (27%) had no BPH. On postoperative evaluation at 3 months, sexual dysfunction scores were significantly improved in 77 patients (88.5%)

**Conclusion:** Patients with bladder stones have a high incidence of sexual dysfunction, particularly those with co-existing LUTS and BPH. About 1/3 patients without BPH had sexual dysfunction and surgical removal of bladder stones significantly improved sexual function and LUTS.

## Take Home Message

Patients with bladder stones have a high incidence of sexual dysfunction, particularly those with co-existing LUTS and BPH. About 1/3 patients without BPH had sexual dysfunction. Surgical removal of bladder stones significantly improved sexual function and LUTS.

## Introduction

Bladder stones are common in the patients with bladder outlet obstruction, either from mechanical obstruction, as in older male patients with benign prostatic hyperplasia (BPH), or functional obstruction, as in patients with a neurogenic bladder. The risk factors for bladder stone include recurrent bladder infections, presence of a foreign body in the bladder, kidney stones, congenital abnormalities of the urinary tract and dehydration (1). Typical symptoms of bladder stones include intermittent painful micturition, terminal hematuria, and lower urinary tract symptoms (LUTS). Common complications of bladder stones include chronic bladder dysfunction and urinary tract infections. However, few studies have evaluated sexual dysfunction in patients with bladder stones. The purpose of this study was to characterize the presence of sexual dysfunction in men with bladder stones. In addition, we assessed the correlates of sexual dysfunction and LUTS in patients with lower urinary tract stones and evaluated the beneficial effects of stone removal on improvement of sexual dysfunction.

## Materials and Methods

This longitudinal prospective observational cohort study was conducted from January 2015 to May 2016. Collection of patient information in this study was approved by Nanfang Hospital at the Southern Medical University Institutional Review Board. All male patients with a diagnosis of bladder stones were enrolled after obtaining written informed consent. All enrolled patients underwent a comprehensive clinical examination prior to surgery, including measurement of blood pressure, heart rate, weight, height, urinalysis, and routine clinical blood measurements. All patients underwent pneumatic lithotripsy for bladder stones and were followed-up at 3, 6, and 12 months after surgery.

**Figure 1:**
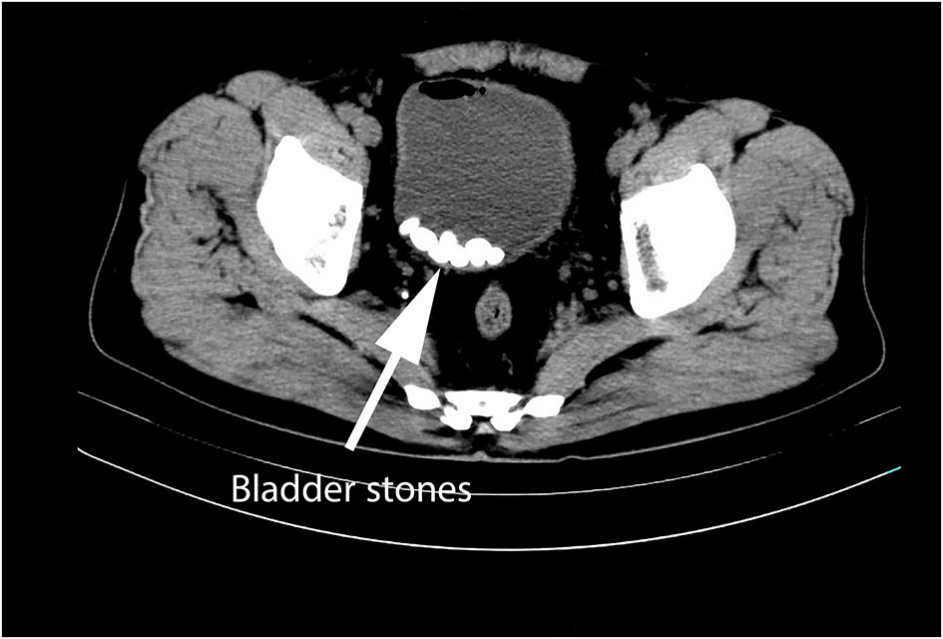
An example of CT image of typical multiple bladder stones.

The number and size of bladder stones were evaluated preoperatively using ultrasound, computed tomography (CT), or cystoscopy, and then determined by postoperative analysis (Figure 1). Urinary tract infection was defined as the presence of ≥ 3 white blood cells per high-power field under a conventional bright field microscope and urine bacteria count >10^5^ per milliliter on urine examination. Urinary frequency, difficulty in micturition, and hematuria were considered as LUTS. All patients underwent endoscopic pneumatic lithotripsy.

Sexual function was evaluated before surgery, and 3, 6, and 12 months after surgery. The International Index of Erectile Function short version (IIEF-5) score, Premature Ejaculation Diagnostic Tool (PEDT) score, and sexual life satisfaction score were used to assess sexual function. The severity of ED was measured with the IIEF short version score (IIEF-5), with a score >21 indicating no ED>21, 17 to <21 mild ED, 12 to <17 mild-moderate ED, 8–11 moderate ED, and <8 severe ED (2). The PEDT was used to assess premature ejaculation. A score ≥ 11 is considered diagnostic of premature ejaculation, 9–10 is an indication for further evaluation, and a score < 9 suggests that premature ejaculation is unlikely (3, 4). Sexual life satisfaction (SLS) is a comprehensive measure of sexual function, measuring the degree to which an individual is satisfied or happy with the sexual aspect of his/her health. SLS was assessed with a single question about the extent to which the respondent was satisfied with his sex life in the past 1 month. The responses were recorded using a 5-point Likert scale (1 = very dissatisfied; 2 = dissatisfied; 3 = fair; 4 = satisfied; 5 = very satisfied) (5, 6). Preoperative assessment of sexual function was performed using the preoperative field questionnaire form. Postoperative assessment was performed during out-patient visits or via telephone follow-up or postal correspondence. In addition, data was collected on the presence of other conditions, including benign prostatic hyperplasia (BPH), neurogenic bladder, infection, foreign body in the bladder, kidney stones, or congenital abnormalities in the urinary tract.

Statistical analysis was performed using the Statistical Package for Social Sciences (SPSS) software for Macintosh (version 20.0; SPSS, Inc.). Data are presented as mean ± variance or as frequency (percentage), as appropriate. Prior to surgery, the sexual function of patients grouped according to the presence of different conditions was analyzed using the two-sample *t*-test. All tests were two-sided and *P* < 0.05 were considered indicative of statistical significance. Preoperative and postoperative sexual function scores at 3, 6, and 12 months were analyzed using repeated measures Analysis of Variance (ANOVA) using a general linear model.

## Results

**Figure 2:**
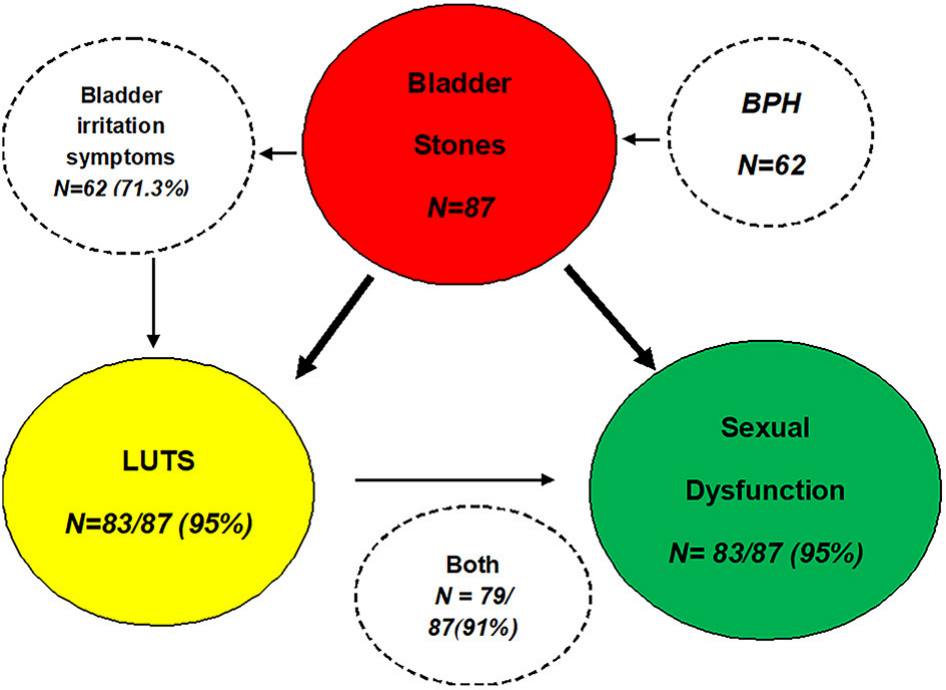
Diagram showing the relationship between sexual dysfunction, bladder stones, and LUTS in the study population.

Ninety patients met criteria to participate in the study. Three patients were lost to follow-up after surgery and were not included in the study. The average age of the 87 patients was 54.7 ± 9.8 years. Sexual dysfunction happened in 83 of 87 patients with bladder stones (95%). Among 83 patients with both bladder stones and sexual dysfunction, 61 patients (73%, age range from 26 to 66 years) had BPH and 22 patients (27%, age range 26 to 65 years) had no BPH. Seventy-nine patients (91%) had both sexual dysfunction and LUTS simultaneously (Figure 2). All patients underwent surgery for bladder stones. None of the patients underwent transurethral resection of the prostate. No patients took medications that significantly affected sexual function.

Fifty-six patients (62.1%) had a single stone, and 33 (37.9%) had multiple stones (range, 2–6). Sixty-one patients (70%) had small stones (diameter < 3 cm) and 26 (29.9%) had larger stones (≥ 3 cm). The maximum diameter of bladder stones was 6.2 cm. Eighty-four patients (96.5%) had LUTS, including urinary frequency, difficulty in micturition, pain or discomfort in the penis or testicles or hematuria. Sixty-eight patients (78.2%) had >3 WBC/high-power field (HPF). Of 68 patients, the urine bacterial count was >10^5^ per milliliter in 34 patients (50%). Sixteen people had urine cultures. Forty-one patients had bladder stones alone, while 46 patients with bladder stones also had kidney or ureter stones (Table 1). All patients had varying degrees of voiding or obstructive symptoms, including poor stream (unimproved by straining), hesitancy (worsened if bladder is very full), terminal dribbling, incomplete voiding, urinary retention, overflow incontinence (occurs in chronic retention), and episodes of near retention.

**Table 1:** Clinical characteristics of the study population.

**Variable**	**All patients (%)**
Age (years)	54.7 ± 9.80
Number of bladder stones	
Single	62.1% (*n* = 54)
Multiple (≥ 2)	37.9% (*n* = 33)
Bladder stone size	
Small (< 3 cm)	70.1% (*n* = 61)
Large (≥ 3 cm)	29.9% (*n* = 26)
Duration of presence of bladder stones	
< 1 year	54.0% (*n* = 47)
≥ 1 year	46.0% (*n* = 40)
LUTS	
Urinary frequency	74.7% (*n* = 65)
Difficulty urinating or pain or	71.3% (*n* = 62)
discomfort in the penis or	
testicles	
No symptoms	3.4% (*n* = 3)
Hematuria	41.4% (*n* = 36)
Urine white blood cells	
< 3/Hp	21.8% (*n* = 19)
≥ 3/Hp	78.2% (*n* = 68)
Bladder stones alone	47.1% (*n* = 41)
Bladder stones combined with upper urinary tract stones	52.9% (*n* = 46)
Bladder stones with BPH	71.3% (*n* = 62)

*Data presented as mean ± standard deviation or as frequency (percentage)*.

*LUTS, lower urinary tract symptoms; Hp, High power microscopic field of view*.

No significant difference was noted in sexual function scores between patients with bladder stones and patients with bladder plus upper urinary tract stones. In addition, no significant difference with respect to sexual function was observed between patients with single bladder stone and multiple stones or between patients with small stones vs. larger stones (*p* > 0.05). Similarly, the sexual function score was similar in patients in whom the time course of illness was <1 vs. ≥ 1 year (*p* > *0.05*). No significant statistical difference was noted in sexual function scores between patients with bladder stones and patients with bladder stones combined with BPH (*p* > 0.05; Table 2).

**Table 2:** Relationship of bladder stones with sexual dysfunction or LUTS.

**Variable**	**All patients**	**IIEF-5 (%)**	**PEDT (%)**	**SLS(%)**
Bladder stones alone	47.1% (*n* = 41)	14.8 ± 4.2	12.9 ± 3.1	3.8 ± 0.8
Bladder stone combined with upper urinary tract stones	52.9% (*n* = 46)	13.7 ± 4.3	12.5 ± 3.0	3.8 ± 1.0
		*p >* 0.05	*p >* 0.05	*p >* 0.05
Bladder stone number				
Single	62.1% (*n* = 54)	14.6 ± 4.35	13.2 ± 3.02	3.9 ± 0.92
multiple	37.9% (*n* = 33)	13.7 ± 4.14	11.8 ± 2.83	3.7 ± 0.85
		*p >* 0.05	*p* = 0.038	*p* > 0.05
Bladder stone size				
Small (< 3 cm)	70.1% (*n* = 61)	13.8 ± 4.14	12.9 ± 2.60	3.8 ± 0.92
Large (r 3 cm)	29.9% (*n* = 26)	15.3 ± 4.49	12.1 ± 3.79	3.8 ± 0.85
		*p* > 0.05	*p* > 0.05	*p* > 0.05
Duration of presence of bladder stones				
< 1 year	54.0% (*n* = 47)	14.7 ± 4.68	12.9 ± 2.95	3.9 ± 0.97
± 1 year	46.0% (*n* = 40)	13.7 ± 3.73	12.4 ± 3.09	3.8 ± 0.80
		*p* > 0.05	*p* > 0.05	*p* > 0.05
Bladder stone combined with BPH	71.3% (*n* = 62)	17.0 ± 4.54	11.6 ± 2.64	4.0 ± 0.73
Bladder stone without BPH	28.7% (*n* = 25)	13.1 ± 3.65	13.1 ± 3.06	3.7 ± 0.94
		*p* > 0.05	*p* > 0.05	*p* = 0.039
**LUTS**				
Urinary frequency	74.7% (*n* = 65)	13.4 ± 4.2	12.8 ± 3.2	3.8 ± 1
No urinary frequency	25.3% (*n* = 22)	16.9 ± 3.5	12.3 ± 2.50	3.9 ± 0.7
		*p* < 0.05	*p >* 0.05	*p > 0.05*
Difficulty urinating or pain or discomfort in the penis or testicles	71.3% (*n* = 62)	14.2 ± 4.3	12.5 ± 3	3.9 ± 0.9
No difficulty urinating	28.7% (*n* = 25)	14.3 ± 4.4	13.1 ± 3	3.6 ± 0.9
		*p >* 0.05	*p >* 0.05	*p >* 0.05
**No LUTS**	3.4% (*n* = 3)	16.7	14	3.3
Hematuria	41.4% (*n* = 36)	13.8 ± 4	13.1 ± 2.76	3.8 ± 0.9
No hematuria	58.9% (*n* = 51)	14.6 ± 4.5	12.4 ± 3.2	3.8 ± 0.9
		*p >* 0.05	*p >* 0.05	*p >* 0.05
WBCs in urine	87			
< 3/hp	21.8% (*n* = 19)	14.5 ± 4.46	13.1 ± 2.81	4.6 ± 1.54
≥ 3/hp	78.2% (*n* = 68)	14.2 ± 4.25	12.6 ± 3.07	3.8 ± 1.02
		*p >* 0.05	*p >* 0.05	*p =* 0.040
Number of bacteria in urine	68			
< 10^5^/mL	50.0% (*n* = 34)	14.8 ± 4.10	12.6 ± 3.62	3.8 ± 0.99
≥ 10^5^/mL	50.0% (*n* = 34)	13.3 ± 4.37	12.7 ± 2.85	3.7 ± 0.83
		*p >* 0.05	*p >* 0.05	*p >* 0.05

*LUTS, lower urinary tract symptoms; IIEF-5, International Index of Erectile Function-5; PEDT, Premature Ejaculation Diagnostic Tool; SLS, Sexual Life Satisfaction Score; WBC, while blood cells; LUTS, lower urinary tract symptoms; IIEF-5, International Index of Erectile Function-5; PEDT, Premature Ejaculation Diagnostic Tool; SLS, Sexual Life Satisfaction Score*.

Patients with LUTS often have sexual dysfunction. In this study, the IIEF-5 scores of patients with urinary symptoms were significantly different from those of patients without urinary symptoms; however, there was no significant difference between the two groups with respect to PEDT and SLS scores. There was no significant difference between patients with and without difficulty in micturition with respect to the IIEF-5, PEDT, and SLS scores (Table 2). Similarly, there was no significant difference between patients with and without hematuria with respect to IIEF5, PEDT, and SLS scores. Although more patients with bladder stones had chronic urinary tract infections (UTI), no significant difference in IIEF-5 and PEDT scores was observed between the UTI and non-UTI groups (*p* > 0.05; Table 2).

**Figure 3:**
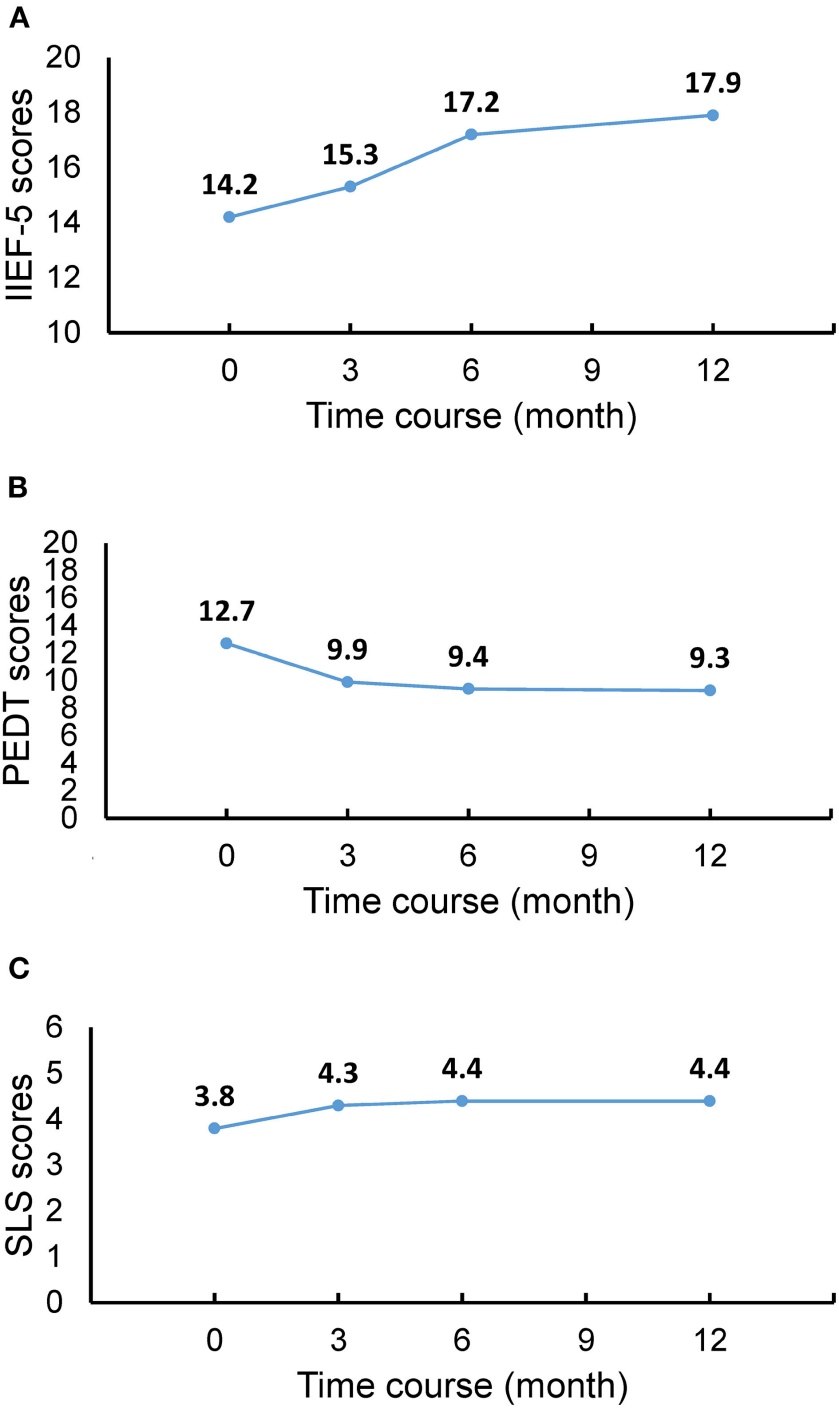
Sexual function recovery after bladder stone ex at various time-points. **(A)** IIEF-5 scores. **(B)** PEDT scores. **(C)** SLS scores. The results show consistent improvement in sexual function after surgical removal of bladder stones. IIEF-5, International Index of Erectile Function-5; PEDT, Premature Ejaculation Diagnostic Tool; SLS, Sexual Life Satisfaction Score.

On preoperative evaluation, the mean IIEF-5 score was 14.2 ± 4.3, the mean PEDT score was 12.7 ± 3, and the mean SLS score was 3.8 ± 0.9 in all of 87 patients. On postoperative evaluation at 3 months, the scores were significantly improved in 77 patients (88.5%)compared to the preoperative scores. There was further improvement in scores at 6 months after surgery (*p* < 0.05). However, there was no significant difference between the 6- and 12-month postoperative evaluation with respect to any of the parameters of sexual function (Figure 3). No significant statistical difference was noted in preoperative and postoperative sexual function scores between patients with bladder stones and patients with bladder stones combined with BPH (*p* > 0.05). Importantly, all 25 (28.7%) patients with the bladder stones alone and 62 (71.3%) with bladder stones with BPH had restored sexual function 12 months after stone removal, indicating the bladder stones contribute to sexual dysfunction, and a combination of bladder stones and BPH increases the rate of sexual dysfunction.

## Discussion

While many complications of bladder stones are well described, sexual dysfunction in these patients has long been ignored. In this study, we demonstrated that there is high prevalence of sexual dysfunction in bladder stone patients either with or without BPH. Moreover, surgical removal of bladder stones significantly improved sexual function. Sexual dysfunction is common in patients with bladder outlet obstruction due to the increasing age and lack of exercise (7). Advanced age and chronic diseases may cause sexual dysfunction, but compared with other studies of patients in the same age group, the incidence of sexual dysfunction in patients in our study is higher. In a recent study of 3,016 adult men, the prevalence of primary premature ejaculation and secondary premature ejaculation was 3 and 4.8%, respectively (8). Out of 88 patients with BPH without bladder stones, 70 (80%) patients had ED, and 60 (68.18%) had poor ejaculation (9). In this study, the prevalence of sexual dysfunction in middle-aged men, i.e., ED and premature ejaculation, was 95.4%, and 72.4% in the patients with bladder stones, respectively. In our study, sexual dysfunction occurred in 98% patient with BPH and bladder stones and in 88% of patients with bladder stones alone Sexual function recovery occurred in 88% patients with either bladder stones alone or the combination. This indicates that sexual dysfunction is highly prevalent in patients with bladder stones compared to the healthy male population. Importantly, patients with bladder stones and no BPH have a high incidence of sexual dysfunction while both bladder stones and BPH leads to an higher rate of sexual dysfunction.

Strengths of this study include objective assessment of impotence before and after stone removal with the IIEF-5 score. There are limitations in our study. This initial study draws on data from a single Medical Center with a limited sample size. The small sample size may have affected the reliability of conclusions, masking the statistical significance of significant differences. The large difference in the results from this and the previously mentioned studies may be attributable to differences in study design, methodology, study population, regions, time frame of investigation, or sampling error. In addition, we did not characterize comorbid conditions in this population.

LUTS are traditionally thought to result from bladder outlet obstruction due to benign prostatic hyperplasia, which gradually leads to increasing symptomatology (10, 11). However, some researchers believe that LUTS are not typically associated with the prostate and that bladder dysfunction is often closely related to LUTS, including overactive bladder, urinary tract structure and functional problems, including urinary stones (12). Primary bladder stones are rare and the cause is unknown. Traditionally, it is believed that the causes of secondary bladder stones include upper urinary tract stones, long-term bed rest, bladder foreign bodies, bladder outlet obstruction (BPH) and bladder dysfunction. Among the 87 cases in this study, there were 22 cases of bladder stones without bladder outlet obstruction, most of which were caused by upper urinary tract stones falling into the bladder. The reason why bladder stones were not discharged from the urethra may be related to the hot and humid weather in Southern China area and dehydration caused by insufficient water intake (1). We speculate that due to dehydration, the urine volume is reduced, and the urine is concentrated. The increased urinary concentration and decreased flow leads to the retention of stones and the increase of stone volume. Bladder mucosa is mechanically rubbed by stones, resulting in cell exfoliation and chronic inflammation of bladder mucosa. This leads to long-term irritation of the mucosa of the bladder neck, which leads to LUTS.

Sexual disorders and their effects on patients were shown to be strongly related to both age and the severity degree of LUTS (13, 14). Epidemiological, pathophysiological and interventional studies have shown a strong positive association between LUTS and ED (14–17). About 70% of patients with LUTS have ED (18). In a study by Rosen et al. (13) the incidence of erectile dysfunction in patients with mild, moderate, and severe LUTS was 43.3, 65.8, and 81.9%, respectively. Treatment of ED with tadalafil (a phosphodiesterase-5 inhibitor) was shown to improve LUTS (19). Similarly, studies have shown a close association between LUTS and premature ejaculation (20–23). In one study, 27% of patients with LUTS were found to be affected by premature ejaculation (21). Terazosin, an alpha-blocker, was shown to alleviate both LUTS and premature ejaculation (22).

LUTS prevalence increases with age. In a population-based survey, 61.2 % of respondents reported at least one symptom of LUTS (23). The prevalence of LUTS in patients with bladder stones has rarely been reported. Up to 95% of patients in this study had LUTS, which suggests that bladder stones may be an important risk factor for LUTS. Conversely, patients with LUTS are at an increased risk for secondary bladder infection due to poor function of the urinary tract, which is one of the causes of bladder stones. Prostate volume is an independent risk factor for bladder stones (24). Therefore, BPH and bladder stones are often mentioned together.

BPH may cause primary bladder stones, and both conditions may lead to LUTS. In turn, LUTS is associated with abnormal sexual function. Patients with both BPH and bladder stones are theoretically more likely to experience LUTS and sexual dysfunction, and our findings support this association. For patients with bladder stones without BPH, bladder stones can be secondary to upper urinary tract stones. There is a high sexual dysfunction rate in patients with BPH. A report by Leliefeld et al. suggested that the relation between BPH and sexual dysfunction is coincidental, unless there are severe symptoms or complications (such as bladder stones or urinary retention) (25). However, few studies have reported sexual function in patients with only bladder stones without lower urinary tract symptoms. The incidence of bladder calculi in patients with BPH is closely associated with preoperative positive urine culture and longer intravesical prostatic protrusion(IPP). Furthermore, IPP may be an independent risk factor for the formation of bladder calculi (26). Benign prostatic hyperplasia (BPH) causes LUTS, and approximately 70% of men with LUTS/BPH have coexisting ED. This prevalence ranges from about 35 to 95% and increases with LUTS severity (7). In our study, the incidence of sexual dysfunction in patients with bladder stones alone (88%) was higher than that in the general population and even higher than that in the BPH patient population. However, the bladder stone patients with no BPH also have a higher sexual dysfunction rate. In addition, stone extraction without surgical correction of BPH still significantly improved sexual function. Compared with patients with bladder stones combined with BPH, the sexual function scores were better, although there was no statistically significant difference. These findings indicate that bladder stones are the primary cause of sexual dysfunction in patients with bladder stones. The mechanism may be that bladder stone cause long-term irritation of bladder neck mucosa, which leads to ED.

The presence and severity of LUTS are risk factors for sexual dysfunction. The causes of sexual dysfunction include psychogenic and organic diseases that generally do not directly cause LUTS symptoms or bladder stones. Since almost all patients included in the study had LUTS, we could not determine whether LUTS directly causes sexual dysfunction. However, the prevalence of sexual dysfunction in patients with bladder stones is higher than that in the general population, which indicates a positive correlation between bladder stones and sexual dysfunction. Bladder stones irritate the bladder wall mucosa and may block the flow of urine, causing signs and symptoms including pain or discomfort in the penis or testicles or LUTS, eventually leading sexual dysfunction.

Analysis of preoperative data suggested no significant association of sexual function with the size and number of bladder stones or the duration of presence of bladder stones (*p* > 0.05 for both). UTI in men may lead to low sexual desire or induce sexual dysfunction (27). However, in this study, the prevalence of sexual dysfunction in patients with chronic UTI was similar to that in patients without UTI. This indicates that although chronic inflammation is more frequent in patients with bladder stones, UTI is not a direct cause of sexual dysfunction in these patients.

In this study, we used endoscopic pneumatic lithotripsy to remove bladder stones. All patients were treated successfully without any serious postoperative complications. In theory, the use of other methods to remove bladder stones may also yield similar results. Indeed, the treatment method should be individualized based on the patient's characteristics and preferences (28–33).

This was a longitudinal prospective cohort study. To the best of our knowledge, no other study has evaluated the relationship between bladder stones and sexual dysfunction. In addition, no study has evaluated whether patients with sexual dysfunction show improvement after surgery for bladder stones. However, we found that LUTS is very common in patients with bladder stones and many patients suffer from sexual dysfunction. Comparison of IIEF-5, PEDT, and sexual life satisfaction scores before and after surgery revealed that surgical treatment of bladder stones can significantly improve the patient's sexual function (*p* < 0.05). This finding indicates that surgical treatment of bladder stones eliminates LUST or bladder irritation symptoms, importantly improves sexual function. Therefore, we have reason to believe that bladder stones are a risk factor for sexual dysfunction, although they do not directly cause the decline in sexual function. Sexual function assessment should be considered in patients with bladder stones.

## Conclusion

In conclusion, sexual dysfunction in patients with bladder stones has been poorly characterized in the past. This study showed that patients with bladder stones have a high incidence of sexual dysfunction. The mechanism of sexual dysfunction in the patients with bladder stone is unclear but LUTS and bladder stimulation might be involved. Surgical removal of bladder stones significantly improved sexual function and LUTS. The presence of the triad of sexual dysfunction, LUTS, and bladder stones provides important insights for investigation of the pathogenesis and treatment of sexual dysfunction in patients with lower urinary tract stones.

## Data Availability Statement

The raw data supporting the conclusions of this article will be made available by the authors, without undue reservation.

## Author Contributions

Z-CG, Z-LW, and Y-AW designed, performed most of the investigation, data analysis, and wrote the manuscript. J-PZ, XW, XL, AB, CD, and MF provided technical assistance. S-CZ and YZ contributed to project design, interpretation of the data and analyses. All of the authors have read and approved the manuscript.

## Conflict of Interest

The authors declare that the research was conducted in the absence of any commercial or financial relationships that could be construed as a potential conflict of interest.

## Publisher's Note

All claims expressed in this article are solely those of the authors and do not necessarily represent those of their affiliated organizations, or those of the publisher, the editors and the reviewers. Any product that may be evaluated in this article, or claim that may be made by its manufacturer, is not guaranteed or endorsed by the publisher.

## References

[1] Zeng G Mai Z Xia S. Prevalence of kidney stones in China: an ultrasonography based cross-sectional study. BJU Int. (2017) 120:109–16. 10.1111/bju.13828 28236332

[2] Rhoden EL Teloken C Sogari PR Vargas SC. The use of the simplified international index of erectile function (IIEF-5) as a diagnostic tool to study the prevalence of erectile dysfunction. Int J Impot Res. (2002) 14:245–50. 10.1038/sj.ijir.3900859 12152112

[3] Symonds T Perelman MA Althof S Giuliano F Martin M May K. Development and validation of a premature ejaculation diagnostic tool. Eur Urol. (2007) 52:565–73. 10.1016/j.eururo.2007.01.028 17275165

[4] Pakpour AH Yekaninejad MS Nikoobakht MR Burri A Fridlund B. Psychometric properties of the Iranian version of the Premature Ejaculation Diagnostic Tool. Sex Med. (2014) 2:31–40. 10.1002/sm2.21 25356299 PMC4184614

[5] World Health Organization. Measuring Sexual Health: Conceptual and Practical Considerations and Related Indicators. Geneva: WHO Press (2010).

[6] Ji F Jiang D Lin X Zhang W Zheng WF Cheng C. Sexual life satisfaction and its associated socio-demographic and workplace factors among Chinese female nurses of tertiary general hospitals. Oncotarget. (2017) 8:54472–7. 10.18632/oncotarget.17664 28903356 PMC5589595

[7] Calogero AE Burgio G Condorelli RA Cannarella R Vignera SL. Epidemiology and risk factors of lower urinary tract symptoms/benign prostatic hyperplasia and erectile dysfunction. Aging Male. (2019) 22:12–9. 10.1080/13685538.2018.1434772 29392976

[8] Gao J Zhang X Su P Liu JS Xia L Yange JJ. Prevalence and factors associated with the complaint of premature ejaculation and the four premature ejaculation syndromes: a large observational study in China. J Sex Med. (2013) 10:1874–81. 10.1111/jsm.12180 23651451

[9] Shao Q Song J Guo YW Lu WC Du LD. Evaluation of sexual function in men with symptomatic benign prostatic hyperplasia. J Chin Androl. (2005) 11:505–7. 10.3969/j.issn.1009-3591.2005.07.007 16078666

[10] Abrams P Cardozo L Fall M. The standardisation of terminology of lower urinary tract function: report from the Standardisation Sub-committee of the International Continence Society. Neurourol Urodyn. (2002) 21:167–78. 10.1002/nau.10052 11857671

[11] Kupelian V Wei JT O'Leary MP Kusek LW Litman HJ Link CL. Prevalence of lower urinary tract symptoms and effect on quality of life in a racially and ethnically diverse random sample: the Boston Area Community Health (BACH) Survey. Arch Intern Med. (2006) 166:2381–87. 10.1001/archinte.166.21.2381 17130393

[12] Chapple CR Wein AJ Abrams P Dmochowski RR Giuliano F Kaplan SA. et al. Lower urinary tract symptoms revisited: a broader clinical perspective. Eur Urol. (2008) 54:563–9. 10.1016/j.eururo.2008.03.109 18423969

[13] Rosen R Altwein J Boyle P Kirby RS Lukacs B Meuleman E. Lower urinary tract symptoms and male sexual dysfunction: the multinational survey of the aging male (MSAM-7). Eur Urol. (2003) 44:637–49. 10.1016/j.eururo.2003.08.015 14644114

[14] Rosen RC Wei JT Althof SE Seftel AD Miner M Perelman MA. Association of sexual dysfunction with lower urinary tract symptoms of BPH and BPH medical therapies: results from the BPH Registry. Urology. (2009) 73:562–6. 10.1016/j.urology.2008.05.034 19167031

[15] McVary KT. BPH: epidemiology and comorbidities. Am J Manag Care. (2006) 12(5 Suppl.):S122–8. 16613526

[16] Kohler TS McVary KT. The relationship between erectile dysfunction and lower urinary tract symptoms and the role of phosphodiesterase type 5 inhibitors. Eur Urol. (2009) 55:38–48. 10.1016/j.eururo.2008.08.062 18783872

[17] Seftel AD de la Rosette J Birt J Porter V Zarotsky V Viktrup L. Coexisting lower urinary tract symptoms and erectile dysfunction: a systematic review of epidemiological data. Int J Clin Pract. (2013) 67:32–45. 10.1111/ijcp.12044 23082930

[18] Oelke M Bachmann A Descazeaud A Emberton M Gravas S Michel MC. EAU guidelines on the treatment and follow-up of non-neurogenic male lower urinary tract symptoms including benign prostatic obstruction. Eur Urol. (2013) 64:118–40. 10.1016/j.eururo.2013.03.004 23541338

[19] Roehrborn CG Egan KB Miner MM Ni X Wong DD Rosen RC. Erectile dysfunction and lower urinary tract symptoms associated with benign prostatic hyperplasia (LUTS/BPH) combined responders to tadalafil after 12 weeks of treatment. BJU Int. (2016) 118:153–60. 10.1111/bju.13406 26765325

[20] Lee JH. Associations between premature ejaculation, lower urinary tract symptoms, and erectile dysfunction in middle-aged Korean policemen. J Sex Med. (2014) 11:1512–18. 10.1111/jsm.12461 24528521

[21] Silangcruz JM Chua ME Morales ML Jr. Prevalence and factor association of premature ejaculation among adult Asian males with lower urinary tract symptoms. Prostate Int. (2015) 3:65–9. 10.1016/j.prnil.2015.03.001 26157771 PMC4494636

[22] Basar MM Yilmaz E Ferhat M Başar H Batislam E. Terazosin in the treatment of premature ejaculation: a short-term follow-up. Int Urol Nephrol. (2005) 37:773–77. 10.1007/s11255-005-3616-4 16362597

[23] Wang Y Hu H Xu K Wang XF Na YQ Kang XP. Prevalence, risk factors and the bother of lower urinary tract symptoms in China: a population-based survey. Int Urogynecol J. (2015) 26:911–9. 10.1007/s00192-015-2626-8 25653032

[24] Jung JH Park J Kim WT Kim HW Kim HJ Hong SW. The association of benign prostatic hyperplasia with lower urinary tract stones in adult men: a retrospective multicenter study. Asian J Urol. (2018) 5:118–21. 10.1016/j.ajur.2017.06.008 29736374 PMC5934505

[25] Leliefeld HH1 Stoevelaar HJ McDonnell J. Sexual function before and after various treatments for symptomatic benign prostatic hyperplasia. BJU Int. (2002) 89:208–13. 10.1046/j.1464-4096.2001.01817.x 11856100

[26] Huang W Cao JJ Cao M Wu HS Yang YY Xu ZM. Risk factors for bladder calculi in patients with benign prostatic hyperplasia. Medicine (Baltimore). (2017) 96:e7728. 10.1097/MD.0000000000007728 28796057 PMC5556223

[27] Pellati D1 Mylonakis I Bertoloni G Fiore C Andrisani A Ambrosinie G. Genital tract infections and infertility. Eur J Obstet Gynecol Reprod Biol. (2008) 140:3–11. 10.1016/j.ejogrb.2008.03.009 18456385

[28] Xun Yang Chen Mingzhen Liang Ping. A novel clinical-radiomics model pre-operatively predicted the stone-free rate of flexible ureteroscopy strategy in kidney stone patients. Front Med (Lausanne). (2020) 7:576925. 10.3389/fmed.2020.576925 33178719 PMC7593485

[29] Ku JH Jung TY Lee JK Park WH Shim HB. Risk factors for urinary stone formation in men with spinal cord injury: a 17-year follow-up study. BJU Int. (2006) 97:790–3. 10.1111/j.1464-410X.2006.05991.x 16536775

[30] Neisius A Wöllner J Thomas C Roos FC Brenner WG Hampel C. Treatment efficacy and outcomes using a third generation shockwave lithotripter. BJU Int. (2013) 112:972–81. 10.1111/bju.12159 24118958

[31] Turney BW Reynard JM Noble JG Keoghane SR. Trends in urological stone disease. BJU Int. (2012) 109:1082–7. 10.1111/j.1464-410X.2011.10495.x 21883851

[32] Herr HW. 'Crushing the stone': a brief history of lithotripsy, the first minimally invasive surgery. BJU Int. (2008) 102:432–5. 10.1111/j.1464-410X.2008.07639.x 18422769

[33] Li Jiali Xun Yang Li Cong. Estimation of renal function using unenhanced computed tomography in upper urinary tract stones patients. Front Med (Lausanne). (2020) 7:309. 10.3389/fmed.2020.00309 32719802 PMC7347744

